# Treatment-Specific Network Modulation of MRI-Guided Focused Ultrasound Thalamotomy in Essential Tremor: Modulation of ET-Related Network by MRgFUS Thalamotomy

**DOI:** 10.1007/s13311-022-01294-9

**Published:** 2022-09-09

**Authors:** Yongqin Xiong, Jiaji Lin, Xiangbing Bian, Haoxuan Lu, Jiayou Zhou, Dekang Zhang, Longsheng Pan, Xin Lou

**Affiliations:** 1 Department of Radiology, Chinese PLA General Hospital/Chinese PLA Medical School, 28 Fuxing Road, Beijing, 100853 China; 2 Department of Neurosurgery, Chinese PLA General Hospital/Chinese PLA Medical School, 28 Fuxing Road, Beijing, 100853 China

**Keywords:** Essential tremor, MR-guided focused ultrasound, Thalamotomy, fMRI, Network

## Abstract

MRI-guided focused ultrasound (MRgFUS) thalamotomy is a novel, effective, and non-invasive treatment for essential tremor (ET). However, the network mediating MRgFUS in treating ET is not precisely known. This study aimed to identify the disease-specific network associated with the therapeutic effects of MRgFUS thalamotomy on ET and investigate its regional characteristics and genetic signatures to gain insights into the neurobiological mechanism of ET and MRgFUS thalamotomy. Twenty-four ET patients treated with MRgFUS thalamotomy underwent resting-state functional MRI at baseline and postoperative 6 months to measure the fractional amplitude of low-frequency fluctuation (fALFF). Ordinal trends canonical variates analysis (OrT/CVA) was performed on the within-subject fALFF data to identify the ET-related network. Genetic functional enrichment analysis was conducted to study the genetic signatures of this ET-related network using brain-wide gene expression data. OrT/CVA analysis revealed a significant ET-related network for which subject expression showed consistent increases after surgery. The treatment-induced increases in subject expression were significantly correlated with concurrent tremor improvement. This network was characterized by increased activity in the sensorimotor cortex and decreased activity in the posterior cingulate cortex. It was correlated with an expression map of a weighted combination genes enriched for mitochondria relevant ontology terms. This study demonstrates that the therapeutic effects of MRgFUS thalamotomy on ET are associated with modulating a distinct ET-related network which may be driven by mitochondria relevant neurobiological mechanism. Quantification of treatment-induced modulation on the ET-related network can provide an objective marker for evaluating the efficacy of MRgFUS thalamotomy.

**Supplementary Information:**

The online version contains supplementary material available at 10.1007/s13311-022-01294-9.

## Introduction

Essential tremor (ET), the most common movement disorder, is characterized by progressive postural and kinetic tremors and can cause significant functional disabilities [1]. Although first-line medications are effective in controlling tremor, approximately 50% patients have medication-refractory tremor [2]. MRI-guided focused ultrasound (MRgFUS) thalamotomy of the ventral intermediate nucleus is a novel and viable treatment option [3]. Reviews have shown that MRgFUS thalamotomy could elicit similar tremor benefits to deep brain stimulation in the treatment of unilateral ET [4, 5]. But compared with deep brain stimulation, MRgFUS is less invasive since it requires no general anesthesia, no burr hole trephination, and no hardware implantation. With these advantages, MRgFUS has gained wide attention. However, the mechanism of MRgFUS thalamotomy in treating ET is not yet completely understood.

Previous studies have revealed that MRgFUS thalamotomy modulated the white matter integrity of the cerebello-thalamo-cortical network of ET patients [6, 7]. Indeed, an association between neuro-network modulations and MRgFUS thalamotomy-induced tremor improvement has been suggested [8]. Identification of the neural network associated with the therapeutic effects of MRgFUS thalamotomy on ET could provide deeper insights into the underlying mechanism and pathophysiology of ET. Group-level statistic method has been extensively used in functional neuroimaging to evaluate network activity, but this approach is largely hampered by the variability in subject and regional activity and is therefore less helpful in prospective single case. A data-driven voxel-based network modeling method has been proposed to address this problem by evaluating network activity through disease-specific spatial covariance pattern. Data-driven spatial covariance analyses of function neuroimaging data have revealed disease-specific networks associated with motor signs, disease progression, and surgical efficacy in Parkinson’s disease [9, 10].

Moreover, approximately 60% of ET patients have a family history [11], suggesting a strong genetic background in ET. Regional gene expressions have been found to be related to the pathological changes in the regions of cerebello-thalamo-cortical circuit in ET patients [12]. Characterizing the potential gene expression linked to the ET-related network mediating MRgFUS thalamotomy may provide valuable insights into the neurobiological mechanism of MRgFUS thalamotomy and ET. The establishment of the Allen Human Brain Atlas (AHBA) provides a key bridge to characterize the genetic architecture of brain imaging phenotypes underlying the neurological disorders and corresponding treatment intervention [13, 14].

In present study, we hypothesized that MRgFUS thalamotomy induced tremor improvement in ET by modulating a disease-specific network, which might be preferentially driven by the neurobiological processes relevant to ET. To test our hypothesis, we (1) applied a within-subject network modeling method to the functional neuroimaging data to identify an ET-related network associated with therapeutic effects of MRgFUS thalamotomy, (2) analyzed the regional characteristics of this network based on atlas- and voxel-wise approaches, (3) used partial least squares (PLS) regression and functional enrichment analysis to characterize its genetic signatures.

## Methods and Materials

### Participants and Study Overview

The study is a pilot clinical trial on the multimodal neuroimaging changes of MRgFUS thalamotomy in treating tremor disorders (ClinicalTrials.gov number: NCT04570046). The study was approved by the Ethics Committee of Chinese PLA General Hospital, and all participants provided written informed consent. The diagnosis of ET was made according to the diagnostic standards proposed by the International Parkinson and Movement Disorder Society, and confirmed by a movement disorder neurologist [15]. Details of the inclusion and exclusion criteria can be found in SI Appendix, Sect. S1.1. Twenty-four ET patients who underwent unilateral MRgFUS thalamotomy were included in present study.

MRgFUS thalamotomy was performed using ExAblate (InSightec, Tirat Carmel, Israel) in a 3-T MRI suite (Discovery 750, GE Healthcare, USA) and tremor score was assessed using the clinical rating scale for tremor (CRST) (SI Appendix, Sects. S1.2 and S1.3) [16]. MRI data were collected using GE Discovery MR750 3.0 T scanner (see SI Appendix, Sect. S1.4 for details of acquisition parameters). The data obtained on preoperative 1 to 3 days was taken as the baseline. The patients were planned to be followed up at 1, 3, and 6 months after treatment to assess clinical and imaging features. Due to the COVID-19 pandemic, however, only 11 out of 24 patients completed multiple follow-up time points as planned, and the data of these patients were used for longitudinal analyses. In addition, 24 age- and gender-matched healthy controls were recruited (16 males and 8 females, 60.50 ± 11.17 years) at baseline.

### Data Preprocessing

The resting-state fMRI data were preprocessed using a graph theoretical network analysis (GRETNA) toolbox (v2.0) [17]. The preprocessing steps included discarding the first five volumes, correcting slice-time and head motion, co-registering and spatially normalizing into MNI EPI brain template. All images were then smoothed with a 4-mm full width at half maximum Gaussian kernel. Further denoising processes included removing the linear drift, and regressing out the Friston-24 motion parameters, white matter, and cerebrospinal fluid signals. No participant had head translation > 3 mm or angular rotation > 3° in any direction. To identify the ET-related network of spontaneous neural activity, we analyzed fMRI data using the fractional amplitude of low-frequency fluctuation (fALFF) approach [18].

### Voxel-Based Network Analysis for MRgFUS Thalamotomy

To identify disease-specific spontaneous neural activity network associated with MRgFUS thalamotomy effects on ET, we used a within-subject voxel-based network approach termed ordinal trends canonical variates analysis (OrT/CVA) [19]. The baseline and postoperative 6-month fALFF data were submitted to OrT/CVA analysis, which yielded an ET-related pattern of fALFF (ETRP-fALFF). The significance of the resulted ETRP-fALFF was evaluated using permutation test (*P* < 0.05, 1000 permutations). A bootstrap resampling procedure (500 re-samplings) was used to evaluate the voxel weight reliability of the ETRP-fALFF (z-map). The ETRP-fALFF was further validated in a leave-one-out design. We also performed OrT/CVA for lesioned and non-lesioned hemisphere fALFF data separately, instead of submitting the data as a whole brain as above, to investigate the hemispheric network of treated and untreated sides (SI Appendix, Sect. S1.5).

The topographic profile rating (TPR) technique was used to the postoperative 1- and 3-month fALFF data available in patients and fALFF data of heathy controls to quantify the corresponding ETRP-fALFF expression. To characterize the longitudinal changes of the ETRP-fALFF expression over time, a linear mixed model was used to analyze the patients’ network expression at baseline and postoperative 1, 3, and 6 months (see SI Appendix, Sect. S1.6 for more details). The effect size of the ETRP-fALFF expression was evaluated using Cohen’s *d* value [20] (see SI Appendix, Sect. S1.6 for more details). Two-sample *t*-tests were performed to compare the network expressions of patients at pre- and post-operation to those of healthy controls to investigate the specificity of the ETRP-fALFF. In order to investigate the clinical relevance of the ETRP-fALFF, spearman rank correlation analyses were performed between the ETRP-fALFF expressions and tremor scores (i.e., hand tremor score, CRST-A score, CRST-B score, CRST-C score, and the CRST total score). A significant threshold of *P* < 0.05 was set.

### Regional Characteristic Analysis of the ETRP-fALFF

In order to clarify the regional characteristic of the ETRP-fALFF, ETRP-fALFF z-maps were mapped to the Brainnetome atlas to quantify the contribution of each region. The regions were ranked according to the absolute regional *z* value and the top 10% regions of interest (ROIs) in the ranked list were identified as having high contribution to the ETRP-fALFF. Spearman rank correlation analysis was conducted to investigate the relationship between the top ROIs and tremor scores. *P* < 0.05 was considered statistically significant. To avoid the limitations of atlas-wise approach, we further analyzed the regional features of ETRP-fALFF based on voxel-wise method [21]. Brain regions that significantly contributed to the ETRP-fALFF were identified at a voxel weight threshold of |z|> 1.96 (*P* < 0.05) with an extent cutoff of 50 voxels. Paired *t*-test was performed to compare the changes of fALFF in these regions between the baseline and postoperative 6 months. Linear mixed model analyses were conducted for the longitudinal changes of fALFF, where Y is the fALFF of each region and X is the vector of time points.

### Genetic Feature Analysis of ETRP-fALFF

The AHBA was introduced to investigate the relationship between gene transcriptional profiles and ETRP-fALFF. The gene expression map contains 20,737 genes based on 58,692 probes of gene expression in 3702 brain samples collected from six adult human brains [22]. The gene expression map was spatially co-registered to the Brainnetome atlas using the abagen toolbox (threshold = 0.5) [23]. As a result, 260 regions of 15,633 genes were extracted. Then, PLS regression was conducted to investigate the fundamental relationships between the ETRP-fALFF z-map and gene expressions measurements for 15,633 genes [24]. Permutation test was used to determine the statistical significance of the variance explained by PLS1 by randomly spinning the response variables 1000 times. The bootstrapping was applied to assess the contribution of each gene to PLS1 by resampling the 360 cortical regions. The ranked list of genes according to the PLS main component (PLS1) was fed into WebGestalt for functional enrichment with 1000 times permutations, with the significant level of false discovery rate (FDR) *q* < 0.05 [25].

### Data Availability

The conditions of our ethics approval do not permit the public archiving of raw data.

## Results

### Demographic and Clinical Details

**Fig. 1:**
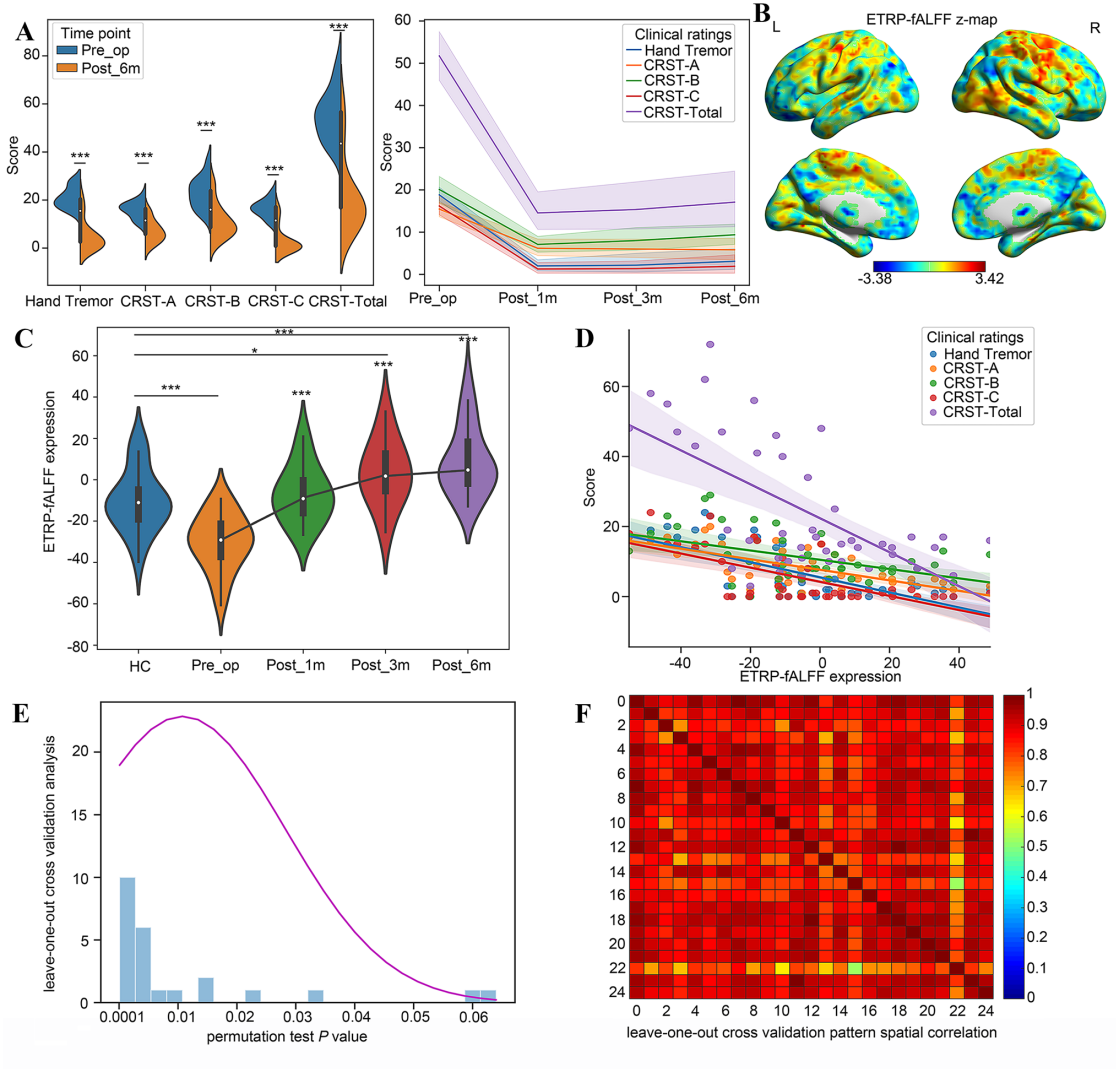
Essential tremor-related network (ETRP-fALFF). **A** Tremor scores were significantly improved by MRgFUS thalamotomy. **B** A significant ET-related network was identified using OrT/CVA. **C** The ETRP-fALFF expression significantly increased after MRgFUS thalamotomy. **D** The ETRP-fALFF expression significantly correlated with tremor scores. **E** and **F** The patterns resulted from each fold of leave-one-out cross validation were highly correlated

A total of 24 patients with ET (16 males) who were followed up at 6 months after MRgFUS thalamotomy were finally included in present study. All patients experienced left thalamotomy. The mean age of the 24 patients was 61.17 years (±11.49) with an average disease duration of 19.50 years. The demographics and clinical characteristics of the participants are summarized in Table 1. MRgFUS thalamotomy resulted in significant improvement in tremor scores at postoperative 6 months: hand tremor score (improvement percentage 78.19%, Cohen *d* = −3.60), the CRST-A score (56.40%, Cohen *d* = −2.49), the CRST-B score (52.92%, Cohen *d* = −1.82), the CRST-C score (82.06%, Cohen *d* = −3.51), and the CRST total score (63.13%, Cohen *d* = −3.22) (all *P* < 0.001, Fig. 1A). The linear mixed model was applied to investigate the dynamic changes of tremor after MRgFUS thalamotomy in 11 patients who were followed up multiple times as planned, and the results showed that the patients obtained immediate and persistent tremor improvement after MRgFUS thalamotomy (Table 2).

**Table 1:** Cohort demographics and clinical data

Criteria	
Patients	24
Gender	
Males	16 (67%)
Females	8 (33%)
Age (years)	
Mean ± SD	61.17 ± 11.49
Range	30–76
Disease duration (years)	
Mean ± SD	19.50 ± 10.48
Range	8–40
Height (cm)	
Mean ± SD	169.25 ± 6.89
Range	155–183
Weight (kg)	
Mean ± SD	72.15 ± 12.58
Range	45–92
Family history	
Yes	19 (79%)
No	5 (21%)
Thalamotomy side	
Left Vim	24 (100%)
Right Vim	0 (0%)
SDR	
Mean ± SD	0.51 ± 0.11
Range	0.35–0.75

*SDR* skull density ratio

**Table 2:** Longitudinal analysis on the tremor ratings after MRgFUS thalamotomy

	Longitudinal analysis			
Items	Linear mixed model	Post hoc		
		*Post hoc*	*P*	Effect size^a^
	F_3, 29.09_ = 207.96 *P* < 0.001	Post_1m < Pre_op	< 0.001	−6.20
Hand tremor		Post_3m < Pre_op	< 0.001	−6.14
		Post_6m < Pre_op	< 0.001	−5.80
	F_3,28.81_ = 42.23 *P* < 0.001	Post_1m < Pre_op	< 0.001	−2.92
CRST-A		Post_3m < Pre_op	< 0.001	−2.98
		Post_6m < Pre_op	< 0.001	−3.04
	F_3,19.28_ = 47.87 *P* < 0.001	Post_1m < Pre_op	< 0.001	−2.44
CRST-B		Post_3m < Pre_op	< 0.001	−2.27
		Post_6m < Pre_op	< 0.001	−2.02
	F_3,18.12_ = 111.73 *P* < 0.001	Post_1m < Pre_op	< 0.001	−3.46
CRST-C		Post_3m < Pre_op	< 0.001	−3.44
		Post_6m < Pre_op	< 0.001	−3.31
	F_3,17.80_ = 150.52 *P* < 0.001	Post_1m < Pre_op	< 0.001	−3.77
CRST-Total		Post_3m < Pre_op	< 0.001	−3.69
		Post_6m < Pre_op	< 0.001	−3.51

*Pre_op* pre-operation, *Post_1m* postoperative 1-month, *Post_3m* postoperative 3 months, *Post_6m* postoperative 6 months

^a^Effect size, evaluated by Cohen *d*

### MRgFUS Thalamotomy Effects on ET-Related Network Expression

A within-subject network modeling technique (i.e., OrT/CVA) was used to analyze the baseline and postoperative 6-month fALFF data. It revealed a significant ET-related spatial covariance pattern of spontaneous neural activity (ETRP-fALFF, Fig. 1B) and quantified the expression of the pattern in each subject at baseline and postoperative 6 months. The expression of the ETRP-fALFF in individuals exhibited a significant increase at 6 months after treatment compared to baseline (*P* = 0.002, permutation test), in that network activity increased with thalamotomy in 22/24 patients. A series of single-case TPR calculation was used to the postoperative 1- and 3-month fALFF data available in patients to quantify the network expression at corresponding time point. A linear mixed model was used to analyze the network expression at baseline and postoperative 1, 3, and 6 months and revealed that the subject expression of the ETRP-fALFF exhibited immediate increase at postoperative 1 month compared to baseline (*P* < 0.001, Cohen *d* = 1.94), and maintained the increasing trend by postoperative 3 months (*P* < 0.001, Cohen *d* = 2.68) and 6 months (*P* < 0.001, Cohen *d* = 3.72) (Fig. 1C). In comparison with the analogous expression of healthy controls, the preoperative ETRP-fALFF expression of patients showed a significant decline (*P* < 0.001, Cohen *d* = 1.40), but slightly increased at 1 month (*P* = 0.50) and significantly increased at 3 (*P* < 0.05) and 6 months (*P* < 0.001) after surgery, indicating the ET-related network was rescued by MRgFUS thalamotomy (Fig. 1C). Leave-one-out strategy was further applied for cross-validation. The results showed that there were significant correlations between the patterns resulting from each leave-one-out fold, which demonstrated the robustness of the ETRP-fALFF (Fig. 1E and F). In addition, baseline and postoperative 6-month hemispheric fALFF data were submitted to OrT/CVA and revealed significant hemispheric ETRP-fALFF for both lesioned and non-lesioned hemisphere (permutation test, *P* < 0.05). But there is no significant difference in the hemispheric ETRP-fALFF expression between lesioned and non-lesioned sides at baseline and at postoperative 6 months (SI Appendix, Fig. S1).

To demonstrate the clinical relevance of the ET-related network, we conducted Spearman rank correlation analyses, exploring the association between ETRP-fALFF expressions and tremor scores. The results showed there were significant negative correlations between ETRP-fALFF expressions and the treated hand tremor score (*Rho* = −0.66), the CRST-A score (*Rho* = −0.66), the CRST-B score (*Rho* = −0.47), the CRST-C score (*Rho* = −0.67), and the CRST total score (*Rho* = −0.63) (all *P* < 0.001, Fig. 1D).

### Topographic Characterization of ET-Related Network

**Fig. 2:**
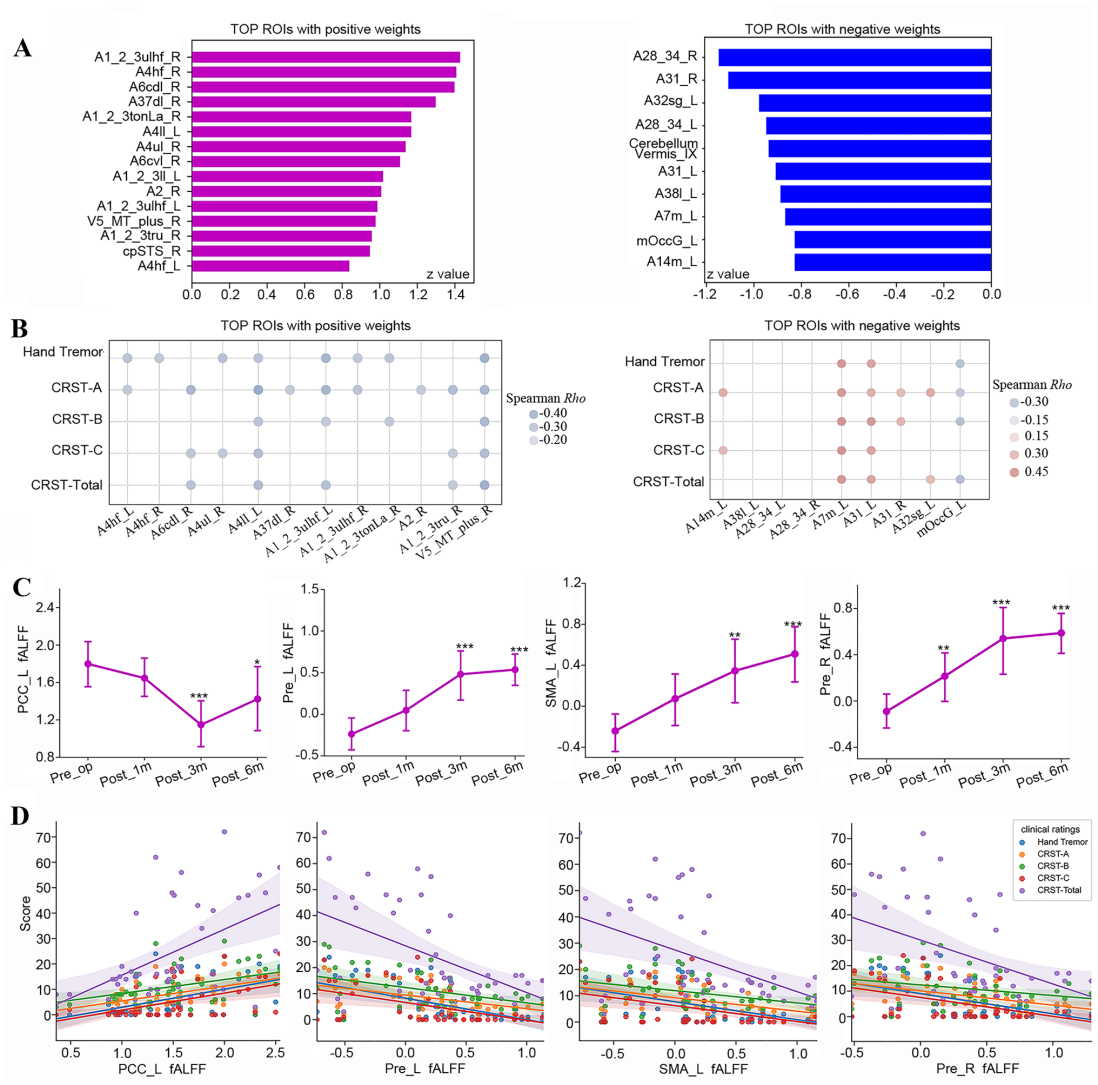
Regional characteristics of the ET-related network. **A** The top 10% ROIs with high weights to ETRP-fALFF identified based on Brainnetome atlas. **B** Correlation analysis results between fALFF in the top 10% ROIs and tremor scores. **C** The longitudinal changes of fALFF in the significant regions identified based on voxel-wise contribution. **D** Significant correlations between fALFF in the significant regions and tremor scores. ROIs, regions of interest

The regional features of ETRP-fALFF based on the Brainnetome atlas showed that the top 10% ROIs of high contributions included the upper limb, head, and face region of bilateral postcentral gyrus (*A1_2_3ulhf_R, A1_2_3ulhf_L*), head and face region of bilateral precentral gyrus (*A4hf_R, A4hf_L*), trunk region of right postcentral gyrus (*A4ul_R*), right caudal dorsolateral area of precentral gyrus (*A6cdl_R*), tongue and larynx region of right postcentral gyrus (*A1_2_3tonLa_R*), right lateral occipital cortex (*V5_MT_plus_R*), right entorhinal cortex of parahippocampal gyrus (*A28_34_R*), right Lc1 of posterior cingulate cortex (*A31_R*), left subgenual area 32 of cingulate gyrus (*A32sg_L*), left medial area of posterior cingulate cortex (*A7m_L*), and left lateral occipital cortex (*mOccG_L*) (Fig. 2A). All the top 10% ROIs are provided in SI Appendix, Table S1. Spearman rank correlation analyses were further performed to investigate the relationship between the fALFF in the top 10% ROIs and tremor scores, and the results showed that there were significant correlations between fALFF in these top regions and tremor ratings (*P* < 0.05) (Fig. 2B).

To avoid the limitations of atlas-wise approach, the topographic characteristics of ETRP-fALFF was also analyzed according to voxel-wise threshold, as in previous study [21]. The results showed that the ETRP-fALFF was characterized by increased functional activity in the bilateral precentral gyrus (Brodmann area 4) and left supplemental motor cortex (Brodmann area 6), but decreased functional activity in the left posterior cingulate cortex and the adjacent precuneus (Brodmann area 31). These results were consistent with the above findings identified using atlas-wise approach. Therefore, mean fALFF in these regions was further extracted and used in the next analyses. In order to investigate the effects of MRgFUS thalamotomy on the brain activity in these significant regions, the paired *t*-test was used to compare the change of fALFF between baseline and postoperative 6 months. Compared with the baseline, at postoperative 6 months, the fALFF significantly decreased in the left posterior cingulate cortex (*P* = 0.001, Cohen *d* = −0.95), but increased in the left precentral gyrus (*P* < 0.001, Cohen *d* = 1.48), left supplemental motor cortex (*P* < 0.001, Cohen *d* = 1.53), and right precentral gyrus (*P* < 0.001, Cohen *d* = 1.68).

To observe the dynamic alterations of the regional brain activity after MRgFUS thalamotomy, we further conducted longitudinal analyses on the fALFF in the significant regions using the linear mixed model. The results showed that fALFF in the regions with positive voxel weights presented significant increase since postoperative 3 months, while fALFF in the regions with negative voxel loadings showed significant decrease since postoperative 3 months (Fig. 2C, Table 3). Spearman rank correlation analysis was conducted to investigate the relationship between fALFF in these regions and tremor scores. The results revealed that fALFF in the left precentral gyrus, left supplemental motor cortex, and right precentral gyrus were negatively correlated with the tremor scores, while fALFF in the left posterior cingulate cortex was positively correlated with tremor scores (*P* < 0.05) (Fig. 2D).

**Table 3:** Linear mixed model analyses on fALFF in the significant regions

	**Longitudinal analysis**			
**items**	**Linear mixed model**	**Post hoc**		
		***Post hoc***	***P***	**Effect size**^**a**^
		Post_1m < Pre_op	0.104	2.11
Pre_L	F_3,22.55_ = 11.44 *P* < 0.001	Post_3m < Pre_op	< 0.001	2.06
		Post_6m < Pre_op	< 0.001	2.23
		Post_1m < Pre_op	0.061	0.97
SMA_L	F_3,19.75_ = 5.86 *P* = 0.005	Post_3m < Pre_op	0.004	1.84
		Post_6m < Pre_op	0.001	2.34
		Post_1m < Pre_op	0.01	1.19
Pre_R	F_3,20.96_ = 12.88 *P* < 0.001	Post_3m < Pre_op	< 0.001	2.42
		Post_6m < Pre_op	< 0.001	2.62
		Post_1m < Pre_op	0.311	-0.34
PCC_L	F_3,20.95_ = 5.47 *P* = 0.006	Post_3m < Pre_op	0.001	-1.48
		Post_6m < Pre_op	0.047	-0.86

*Pre_L* left precentral gyrus, *SMA_L* left supplemental motor cortex, *Pre_R*, right precentral gyrus, *PCC_L* left posterior cingulate cortex

^a^Effect size, evaluated by Cohen *d*

### Signaling Pathway Underlying ET-Related Network

Curious about the signaling pathway underlying the ET-related network, we introduced the AHBA database to investigate the relationship between brain-wide gene expression and ETRP-fALFF. PLS regression was performed to identify gene expression that was highly correlated with the ETRP-fALFF *z*-map. The PLS1 explained 10% of the variance in the ETRP-fALFF *z*-map, which was significant more than expected by chance (*P* < 0.001, SI Appendix, Fig. S2) but insignificant after spatial autocorrection correction (see SI Appendix, Sect. S1.7 for more details). PLS1 gene expression weighted map was positively correlated with ETRP-fALFF z-map (*r* = 0.32, *P* < 0.001). Then, the PLS1 weighted genes were functionally annotated using gene set enrichment analysis (GSEA) to further identify their neurobiological features.

**Fig. 3:**
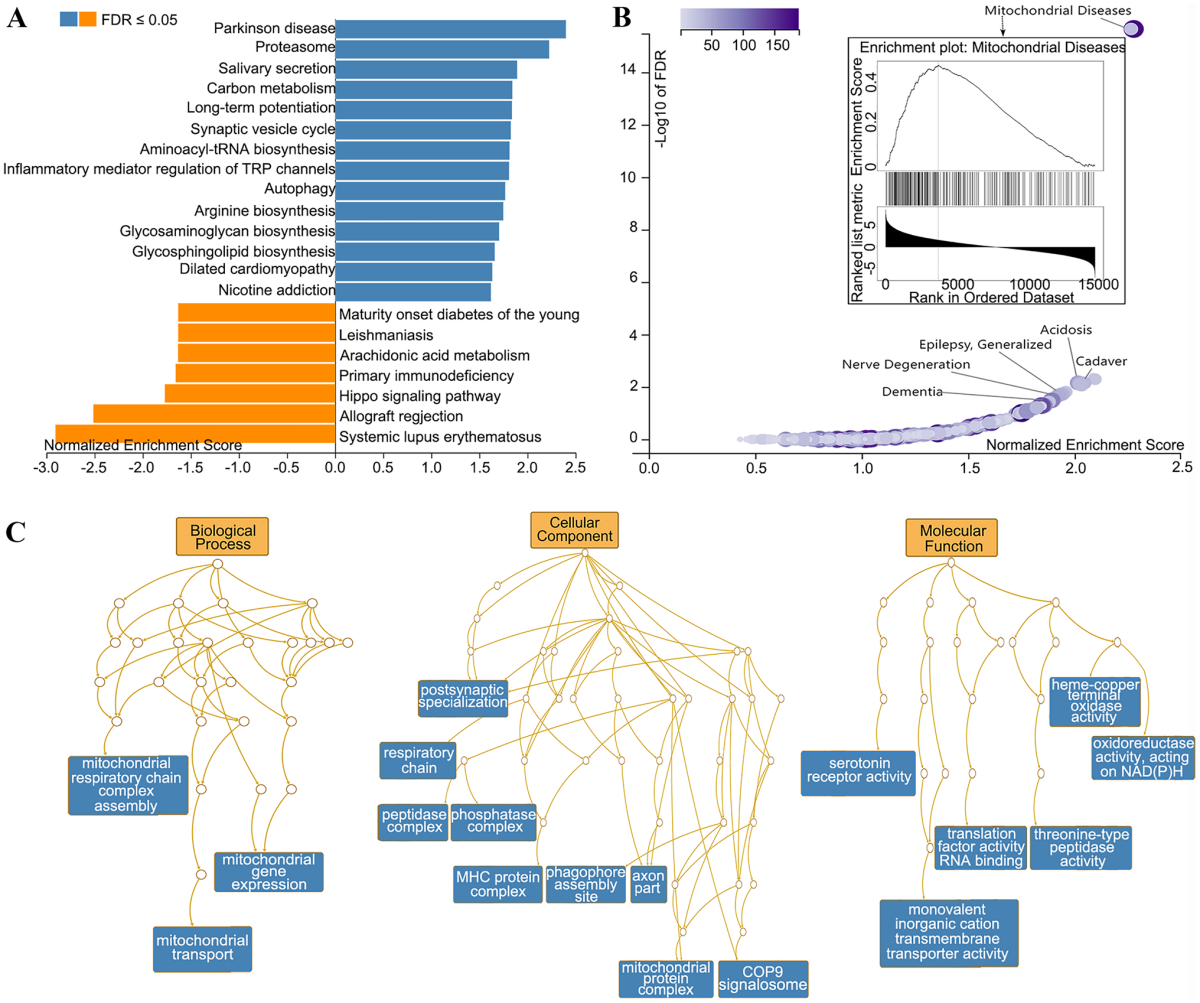
Gene functional enrichment analysis. **A** Significant results of KEGG pathway enrichment analysis. **B** Significant results of GLAD4U disease enrichment analysis. **C** Directed acyclic graph of GO terms enrichment analysis

KEGG pathway analysis revealed that the PLS1 weighted genes were most significantly enriched in the terms related to Parkinson’s disease (KEGG: hsa05012) (Fig. 3A). Both OMIM and GLAD4U disease analyses showed that PLS1 weighted genes were enriched in the terms related to mitochondrial complex I deficiency (OMIM: 252,010) and mitochondrial disease (GLAD4U: PA447172), respectively (Fig. 3B). We further tested the PLS1 gene set for significant Gene Ontology (GO) enrichment of biological processes, cellular component, and molecular function (Fig. 3C). Most of the enriched biological processes were involved in mitochondrial respiratory chain complex assembly (GO:0,033,108), mitochondrial gene expression (GO:0,140,053), and mitochondrial transport (GO:0,006,839) (all FDR *q* < 0.05). The majority of the cellular components focused on respiratory chain (GO:0,070,469) and mitochondrial protein complex (GO:0,098,798). The enriched biological processes included heme-copper terminal oxidase activity (GO:0,015,002), oxidoreductase activity acting on NAD(P)H (GO:0,016,651), and serotonin receptor activity (GO:0,008,135). All significant GO enrichment results are listed in SI Appendix, Tables S2–S4.

## Discussion

The study identified a characteristic and quantifiable essential tremor-related network, i.e., the ETRP-fALFF. MRgFUS thalamotomy induced sustained increase of the ETRP-fALFF expression, which was correlated with clinical improvement in the tremor scores. The ETRP-fALFF was characterized by increased spontaneous neural activity in the bilateral sensorimotor cortex, and decreased activity in the left posterior cingulate cortex. Analyzed with prior brain-wide gene expression data, the ETRP-fALFF was found to be correlated with a spatial expression map of a weighted combination of genes which were enriched in neurobiologically relevant GO terms and KEGG pathways.

### ET-Related Network Mediates the Effects of MRgFUS Thalamotomy

Although the mechanism of MRgFUS thalamotomy for treating ET remains unclear, accumulating evidence suggests that it is related to the modulation of disease-related abnormal network [26, 27]. Therefore, quantification of the network changes induced by MRgFUS thalamotomy could not only provide objective measures for the effects of MRgFUS thalamotomy, but also give new insights into the pathophysiology of ET. The OrT/CVA approach is a within-subject voxel-based network modeling technique designed to identify the specific disease-related spatial covariance pattern, for which subject expression presents consistent change on an individual subject basis. Therefore, it is more helpful in prospective clinical applications with individual subject than in traditional group-level statistics. For example, Mure et al. identified a distinct Parkinson’s disease tremor-related metabolic pattern using OrT/CVA and found that treatment-mediated changes in the expression of this pattern induced by deep brain stimulation could evaluate the therapeutic effects of this intervention on Parkinson’s tremor [9]. In present study, we identified a distinct ET-related network (ETRP–fALFF) using OrT/CVA. The expressions of ETRP-fALFF significantly increased following MRgFUS thalamotomy. The elevated network expression might be closely related to the brain plasticity which is a dynamic and continuous process. Our previous study found that the regional structural network had a significant and long-term neuroplasticity changes following MRgFUS thalamotomy [14]. Such structural alterations might be accompanied by functional alterations. Longitudinal investigations further validated the stability and quantitative utility of the ETRP-fALFF. The clinical relevance of the ETRP-fALFF is supported by the significant correlation seen between clinical relief in the tremor scores and the modulation of the ETRP-fALFF after MRgFUS thalamotomy. This suggests that the clinical benefit achieved following MRgFUS thalamotomy is likely to be mediated by the modulation of ETRP-fALFF. In addition, we found that there was a shift towards normality of ETRP-fALFF expression in response to MRgFUS thalamotomy. In aggregate, the findings indicate that MRgFUS thalamotomy-induced improvement of tremor is associated with the ET-related network, which could serve as an objective quantitative biomarker for the therapeutic effects of MRgFUS thalamotomy.

### Sensorimotor Cortex Contributes Most to ET-Related Network

ETRP-fALFF topography is characterized by the key regions of sensorimotor network and default mode network, including sensorimotor cortex, supplemental motor cortex, and posterior cingulate cortex. The sensorimotor cortex and supplemental motor cortex present significantly positive contribution to the ETRP-fALFF, while posterior cingulate cortex shows negative contribution. MRgFUS thalamotomy induced an increase of spontaneous neural activity in the regions with positive contribution, but a decrease of activity in the regions with negative contribution (Fig. 2D). The findings indicate that the disturbance on the link between the default mode network and sensorimotor network might play a major role in the development of ET. The opposite changes after MRgFUS thalamotomy might indicate a re-establishment of the relationship between the default mode network and sensorimotor network, thus regaining of the ability to coordinate the motor and non-motor network. There were strong correlations between the regulation of MRgFUS thalamotomy on the spontaneous neural activity in these key regions and the clinical improvement induced by this procedure, which further supported the clinical significance of these key regions.

On the one hand, previous studies have demonstrated that the sensorimotor cortex and supplemental motor cortex play an important role in the generation of ET [28, 29]. More recently, it has been shown that MRgFUS thalamotomy led to a decrease of fractional anisotropy in the ipsilateral sensorimotor subcortical white matter in ET patients [6]. These previous reports further support the critical role of the sensorimotor cortex in the ET-related network. On the other hand, we found that the hub center of default mode network (posterior cingulate cortex) showed a significant negative contribution to the ET-related network. Similarly, previous studies have reported that the patients with ET had decreased regional homogeneity in the posterior cingulate cortex and interconnectivity between the sensorimotor and default mode network in comparison with healthy controls [26, 30, 31]. These studies often related the abnormality of default mode network to the poor cognitive performance in ET, but the patients in some of these studies had no cognitive impairment. The role played by the default mode network in the pathogenesis of ET remains unclear. The default mode network, a task-negative network, is activated during resting-state of the brain but deactivated when performing a particular task [32]. There is an anti-correlation between the default mode network and sensorimotor network in young heathy adults at resting-state of the brain, which could segregate the incompatible networks to prevent mutual interference [33]. During the execution of the motor task, the activity of default mode network decreases, the activity of sensorimotor network increases, and the strength of their anti-correlation decreases [34]. But this anti-correlation decrease is reversed in aged people both at resting state and during motor action [33]. The disturbance of this anti-correlation has been proposed to contribute to the motor disturbance of aged people [35, 36]. In current study, contrary to the increase activity of the sensorimotor cortex after MRgFUS thalamotomy, the spontaneous neural activity of the posterior cingulate cortex decreased after surgery in ET. These findings suggest that the impairment of the default mode network-sensorimotor network link might be responsible for the generation of ET, and MRgFUS thalamotomy might re-establish this link to control tremor. This is worth further research.

### Mitochondria-Related Signaling Pathway Drives ET-Related Network

The results of genetic analyses indicate that the ET-related network associated with MRgFUS thalamotomy effects may be driven by mitochondria-related neurophysiological processes. On the one hand, the identified GO enrichment analysis results were primarily related to mitochondria. Previous studies have indicated that mitochondrial dysfunction might be one of the causative factors of ET. Yoo et al. have revealed that ET patients have large deletions within several regions of mitochondrial DNA, and suggested that ET is a disease characterized by showing mitochondrial DNA multicomplex deficiency [37]. Gulsuner et al. have found that the mitochondrial serine protease HTRA2 p.G399S was responsible for the pathogenesis of ET, which was previous reported to cause mitochondrial dysfunction [38]. On the other hand, the functional enrichment pathway analysis showed that the PLS1-identified genes were enriched in the Parkinson’s disease KEGG. Indeed, a large amount of evidence about the link between ET and Parkinson’s disease has been reported, including epidemiologic features, phenomenological characteristics, pathological changes, and pathogenetic signatures [39]. In addition, previous findings have revealed that mitochondrial dysfunction plays a critical role in the pathogenesis of Parkinson’s disease [40, 41]. In turn, these findings further supported that the ET-related network identified in present study might be driven by mitochondria-related signaling pathway.

### Limitations

Several considerations must be acknowledged when interpreting these results. First, this study reported the clinical and network changes at postoperative 6 months. Further studies are needed to observe the long-term changes. Second, only approximately half of patients completed multiple follow-up time points as planned primarily due to the COVID-19 pandemic. Further longitudinal studies with a larger sample size are required to replicate our findings. Third, the whole-brain genome expression data were obtained from the participants without essential tremor and were not matched in age and gender. The results from these data might be biased by these variations.

## Conclusion

This study established a distinct ET-related network associated with therapeutic effects of MRgFUS thalamotomy. Quantification of MRgFUS thalamotomy-induced changes in the ET-related network can provide an objective evaluation of the clinical efficacy of this procedure. Furthermore, regional characteristic and mitochondria relevant signal pathways signatures of ET-related network provide new insights into neurobiological mechanisms underlying the effectiveness of MRgFUS thalamotomy and the pathophysiology of ET.

## Supplementary Information

Below is the link to the electronic supplementary material.

Supplementary file1 (DOCX 1293 KB)

Supplementary file2 (PDF 496 KB)

Supplementary file3 (PDF 497 KB)

Supplementary file4 (PDF 496 KB)

Supplementary file5 (PDF 499 KB)

Supplementary file6 (PDF 498 KB)

Supplementary file7 (PDF 495 KB)

Supplementary file8 (PDF 498 KB)

Supplementary file9 (PDF 497 KB)

## Abbreviations

AHBA: Allen Human Brain Atlas
CRST: Clinical rating scale for tremor
ETRP-fALFF: ET-related spatial covariance pattern of fALFF
FDR: False discovery rate
fALFF: Fractional amplitude of low-frequency fluctuation
GSEA: Gene set enrichment analysis
GO: Gene Ontology
KEGG: Kyoto Encyclopedia of Genes and Genomes
rsfMRI: Resting-state function MRI
MRgFUS: MR-guided focused ultrasound
OMIM: Online Mendelian Inheritance in Man
OrT/CVA: Ordinal trends canonical variates analysis
PLS: Partial least squares
