# Supplementary material for: Treatment-Specific Network Modulation of MRI-Guided Focused Ultrasound Thalamotomy in Essential Tremor: Modulation of ET-Related Network by MRgFUS Thalamotomy
Source: Neurotherapeutics. 2022 Sep 9;19(6):1920–31. doi: 10.1007/s13311-022-01294-9 (PMC9462640; doi:10.1007/s13311-022-01294-9)
Supplement: Supplementary file 1 — Supplementary file1 (DOCX 1293 KB) [file 13311_2022_1294_MOESM1_ESM.docx]

# Supplemental Information for “Treatment-Specific Network Modulation of MRI-guided Focused Ultrasound Thalamotomy in Essential Tremor”

**1. Methods**

**1.1 Inclusion and Exclusion Criteria**

**Inclusion Criteria.** 1) age 22 years or older. 2) medication-refractory tremor: a therapeutic dose of at least two medications did not suppress tremor or intolerant side effects developed as the medication dose is titrated. 3) following the 1-month medication stability period. 4) Vim can be target by the ExAblate device. 5) postural or intention tremor severity score in the dominant hand/arm ≥ 2 (measured by the CRST while stable on medication). 6) significant disability due to ET: any one of CRST-C items ≥ 2 (item #16 to item 23). 7) able to communicate sensations during treatment. 8) study criteria have been agreed upon by two investigators.

**Exclusion Criteria.** 1) unstable cardiac status, severe hypertension, severely impaired renal function, and any other life-threatening systemic disease. 2) ethanol or substance abuse. 3) contraindications for MR imaging, or intolerance or allergies to the MRI contrast agent. 4) history of abnormal bleeding and/or coagulopathy, or risk factors for hemorrhage. 5) active infections, or history of immunocompromise. 6) any other neurodegenerative diseases, cerebrovascular diseases, brain tumors, seizures, or other brain diseases. 7) significant cognitive impairment and mood disorders. 8) history of psychiatric diseases. 9) legal incapacity or an inability to communicate. 10) unable or unwilling to tolerate prolonged stationary supine position during treatment. 11) history of deep brain stimulation or a prior stereotactic ablation. 12) pregnancy or lactation. 13) an overall Skull Density Ratio (SDR) ≤0.35. 14) any conditions that, in the judgment of the investigator, would preclude participation in this study.

**1.2 MRgFUS procedure**

MRgFUS thalamotomy was performed using ExAblate (InSightec, Tirat Carmel, Israel) in a 3-T MRI suite (Discovery 750, GE Healthcare, Milwaukee, WI, USA). Only a unilateral ablation was performed in this study, targeting the ventral intermediate nucleus of the thalamus (Vim) contralateral to the most severely affected extremity.

The patient was first prepared by carefully shaving their head and examining for any scalp scars or other scalp lesions on the day before the procedure. An MRI-compatible stereotactic head frame was applied under local anesthesia. An elastic membrane bag was placed around the patient’s head and fitted to the scalp. The patient was positioned supine and headfirst on the MR/ExAblate therapy table, with the headframe secured in place and the membrane sealed to the ExAblate transducer. The cranium and the transducer (within the elastic membrane) space was filled with circulating degassed and cooled water with care to avoid air bubbles to keep chilled throughout the procedure.

Intraoperative T2 weighted MR images were acquired to identify the target area and plan the treatment. The preoperative CT and MRI image datasets were coregistered and fused to the T2 weighted MR images to help determine the target area and the safe sonication pathway. The initial target coordinates for Vim thalamotomy are anterior to the posterior commissure by 25% of the inter-commissural distance and 14mm lateral to the midline line (11.5mm lateral to the third ventricle wall in cases with an enlarged third ventricle).

A series of sequentially titrated acoustic energy sonications were performed in three stages under the real time monitor of MR thermometry: aligning the ultrasound focus (40 to 45°C), verifying the target coordinates (46 to 50 °C) and ablating the target tissue (approximately 51 to 60°C). The patient was examined by the neurosurgeon during and after each sonication for neurologic signs and symptoms. The procedure lasts approximately 4 hours inside of the MRI. Once the treatment is complete, the headframe is removed. Refer to previous studies for more details of the procedure[1, 2].

**1.3 Tremor evaluation**

Tremor assessments were performed using the Clinical Rating Scale for Tremor (CRST) [3]. The CRST rates the tremor location and severity (Part A), the specific motor tasks/function (Part B), and the functional disabilities resulting from tremor (Part C). The tremor subscore in the treated hand (contralateral to the thalamotomy) was derived from the CRST-A (the item #5) and the CRST-B (the item #11 to item #15, item #11 will be taken into account only for dominant hand). The treated hand tremor subscore ranges from 0 to 32 when the dominant hand is treated and 0 to 28 when the nondominant hand is treated. Higher scores indicate more severe tremor. The improvement percentage was calculated according to the formula: (baseline score - follow-up)/baseline score × 100.

**1.4 MRI data acquisition**

MRI data were collected using GE Discovery MR750 3.0 T scanner (GE Healthcare, Milwaukee, WI, USA). Participants were instructed to lie supine in the scanner, keep as still as possible with their eyes closed, and remain awake but not think specifically. Earplugs were used to reduce the scanner noise. Tight but comfortable foam padding were placed around participants’ head to minimize head motion. The resting-state fMRI (rsfMRI) images were acquired for 180 volumes with an echo planar imaging (EPI) sequence (repetition time = 2000.0 ms, echo time = 30 ms, flip angle (FA) = 90°, field of view (FOV) = 240 mm × 240 mm, matrix size = 64 × 64, slice thickness 3.5 mm, slice gap 0.5 mm, and 36 interleaved slices).

**1.5 Hemispheric voxel-based network analysis for MRgFUS thalamotomy**

The baseline and postoperative 6-month lesioned and non-lesioned hemisphere fALFF data were separately submitted to OrT/CVA to explore hemispheric ETRP-fALFF. The significance of the resulted hemispheric ETRP-fALFF was also evaluated using permutation test (*P* < 0.05, 1000 permutations). Similarly, bootstrap resampling procedures (500 re-samplings) were used to evaluate the voxel weight reliability of the hemispheric ETRP-fALFF. Baseline and postoperative 6-month lesioned hemispheric ETRP-fALFF expression were compared to the non-lesioned hemispheric ETRP-fALFF expression of the corresponding time point using two-sample *t*-test to explore if there is any difference on network score between lesioned and non-lesioned sides before and after surgery.

**1.6 Linear mixed model analysis and effect size evaluation**

A linear mixed model was used to characterize the longitudinal changes of the ETRP-fALFF expression over time:

$$Y_{ij}=\gamma+b_{i}+X_{ij}\beta+ \varepsilon_{ij}$$

where Y*_ij_* is the ETRP-fALFF expression from the *j*th scan of the *i*th patient (*i* ≤ 11, *j* ≤ 4, up to four scans including baseline, and 1, 3 and 6 months after thalamotomy); γ is the intercept item common to all patients; b*_i_* is the unique random intercept for *i*th patient; X*_ij_* is the vector of time points; β represents the fixed effects; ε*_ij_* is the residual error. The restricted maximum likelihood method was used to estimate the mode parameters. The model was considered significant for *P* < 0.05. In the post hoc analysis, pairwise comparisons were performed between the postoperative ETRP-fALFF scores obtained at each time point and the preoperative ones. Statistical comparisons were considered significant if *P* < 0.017 (Bonferroni corrections for three comparisons, *P* = 0.05/3).

The effect size of ETRP-fALFF expression was evaluated by Cohen’s *d* value according to the following formula:

$$effect size=\frac{{Mean}_{post}-{Mean}_{pre}}{\mathrm{SD}_{pre}}$$

where Mean*_pre_* and Mean*_post_* represent the mean values before and after surgery. SD is the standard deviation before treatment. Only preoperative standard deviation was used here because it was assumed to provide an unbiased estimate of σ not being influenced by the intervention[4].

**1.7 Spatial Permutation Test**

Considering the spatial autocorrelation (SA) amongst the regions of the chosen parcellation, a regional spatial permutation test was used to validate the correlation between the ETRP-fALFF z-map and genome-wide expression map. The BrainSMASH toolbox (<https://github.com/murraylab/brainsmash>) [5] was used to generate 1000 surrogate maps with SA matched to SA of the target brain map through variogram. Then the 1000 SA-preserving surrogate maps were input into the spatial permutation test to determine the statistical significance of the variance explained by PLS1.

# Supplemental Tables

**Table S1. Top 10% ROIs contributed to ETRP-fALFF based on the Brainnetome atlas.**

| **Region** | **Full name** | **z-value** |
| --- | --- | --- |
| A1_2_3ulhf_R | area 1/2/3(upper limb, head and face region) of right postcentral gyrus | 1.43 |
| A4hf_R | area 4(head and face region) of right precentral gyrus | 1.41 |
| A6cdl_R | caudal dorsolateral area 6 of right precentral gyrus | 1.40 |
| A37dl_R | dorsolateral area 37 of right middle temporal gyrus | 1.30 |
| A1_2_3tonLa_R | area 1/2/3(tongue and larynx region) of right postcentral gyrus | 1.17 |
| A4ll_L | area 4, (lower limb region) of left paracentral lobule | 1.17 |
| A28_34_R | area 28/34 (entorhinal cortex) of right parahippocampal gyrus | -1.15 |
| A4ul_R | area 4(upper limb region) of right precentral gyrus | 1.14 |
| A31_R | area 31 (Lc1) of right precuneus | -1.11 |
| A6cvl_R | caudal ventrolateral area 6 of right precentral gyrus | 1.11 |
| A1_2_3ll_L | area1/2/3 (lower limb region) of left paracentral lobule | 1.03 |
| A2_R | area 2 of right postcentral gyrus | 1.01 |
| A1_2_3ulhf_L | area 1/2/3(upper limb, head and face region) of left postcentral gyrus | 0.99 |
| V5_MT_plus_R | right lateral occipital cortex | 0.98 |
| A32sg_L | subgenual area 32 of left cingulate gyrus | -0.98 |
| A1_2_3tru_R | area1/2/3(trunk region) of right postcentral gyrus | 0.96 |
| A28_34_L | area 28/34 (entorhinal cortex) of left parahippocampal gyrus | -0.95 |
| cpSTS_R | right caudoposterior superior temporal sulcus | 0.95 |
| Cerebellum_Vermis_IX | Cerebellum Vermis IX | -0.95 |
| A31_L | area 31 (Lc1) of left precuneus | -0.91 |
| A38l_L | lateral area 38 of left superior temporal gyrus | -0.89 |
| A7m_L | medial area 7(PEp) of left precuneus | -0.88 |
| A4hf_L | area 4(head and face region) of left precentral gyrus | 0.84 |
| mOccG_L | left middle occipital gyrus | -0.84 |
| A14m_L | medial area 14 of left orbital gyrus | -0.83 |

**Table S2: Significant Gene Ontology terms of biological processes for PLS1 weighted genes.**

| **Gene Set** | **Description** | **Size** | **NES** | **P value** | ***FDR q* value** |
| --- | --- | --- | --- | --- | --- |
| GO:0033108 | mitochondrial respiratory chain complex assembly | 88 | 2.31 | <0.001 | <0.001 |
| GO:0140053 | mitochondrial gene expression | 158 | 2.09 | <0.001 | <0.001 |
| GO:0099132 | ATP hydrolysis coupled cation transmembrane transport | 48 | 2.10 | <0.001 | <0.001 |
| GO:0009141 | nucleoside triphosphate metabolic process | 262 | 2.11 | <0.001 | <0.001 |
| GO:0072512 | trivalent inorganic cation transport | 32 | 2.01 | <0.001 | 0.0014 |
| GO:1903008 | organelle disassembly | 93 | 2.00 | <0.001 | 0.0016 |
| GO:0106027 | neuron projection organization | 79 | 1.82 | <0.001 | 0.013 |
| GO:0099003 | vesicle-mediated transport in synapse | 188 | 1.80 | <0.001 | 0.014 |
| GO:0006839 | mitochondrial transport | 222 | 1.79 | <0.001 | 0.015 |
| GO:0034765 | regulation of ion transmembrane transport | 353 | 1.76 | <0.001 | 0.017 |
| GO:0072522 | purine-containing compound biosynthetic process | 230 | 1.74 | <0.001 | 0.020 |
| GO:0072524 | pyridine-containing compound metabolic process | 132 | 1.70 | 0.0011 | 0.028 |
| GO:0019098 | reproductive behavior | 24 | 1.68 | 0.0089 | 0.032 |
| GO:0061614 | pri-miRNA transcription by RNA polymerase II | 36 | -2.01 | <0.001 | 0.039 |
| GO:0007034 | vacuolar transport | 125 | 1.64 | <0.001 | 0.039 |
| GO:1990089 | response to nerve growth factor | 48 | 1.63 | 0.011 | 0.041 |
| GO:0042430 | indole-containing compound metabolic process | 16 | 1.63 | 0.0089 | 0.042 |
| GO:0097503 | sialylation | 18 | 1.63 | 0.015 | 0.042 |
| GO:0030705 | cytoskeleton-dependent intracellular transport | 161 | 1.62 | <0.001 | 0.044 |
| GO:0150076 | neuroinflammatory response | 32 | -1.91 | 0.0035 | 0.048 |
| NES: normalized enrichment score. | | | | | |

**Table S3. Significant Gene Ontology terms of cellular component for PLS1 weighted genes.**

| **Gene Set** | **Description** | **Size** | **NSE** | ***P* value** | ***FDR q* value** |
| --- | --- | --- | --- | --- | --- |
| GO:0070469 | respiratory chain | 86 | 2.59 | <0.001 | <0.001 |
| GO:0098798 | mitochondrial protein complex | 248 | 2.55 | <0.001 | <0.001 |
| GO:0042611 | MHC protein complex | 18 | -2.31 | <0.001 | <0.001 |
| GO:1905368 | peptidase complex | 85 | 1.98 | <0.001 | <0.001 |
| GO:0000407 | phagophore assembly site | 30 | 1.77 | 0.0028 | 0.0076 |
| GO:0099572 | postsynaptic specialization | 321 | 1.71 | <0.001 | 0.0094 |
| GO:0033267 | axon part | 335 | 1.73 | <0.001 | 0.0096 |
| GO:1903293 | phosphatase complex | 47 | 1.721 | 0.0027 | 0.0098 |
| GO:0005874 | microtubule | 344 | 1.6 | <0.001 | 0.021 |
| GO:0036019 | endolysosome | 17 | 1.60 | 0.028 | 0.023 |
| GO:0008180 | COP9 signalosome | 34 | 1.56 | 0.032 | 0.031 |

**Table S4. Significant Gene Ontology terms of molecular function for PLS1 weighted genes.**

| **Gene Set** | **Description** | **Size** | **NSE** | ***P* value** | ***FDR* *q* value** |
| --- | --- | --- | --- | --- | --- |
| GO:0015002 | heme-copper terminal oxidase activity | 22 | 2.14 | <0.001 | <0.001 |
| GO:0015077 | monovalent inorganic cation transmembrane transporter activity | 285 | 2.03 | <0.001 | 0.0026 |
| GO:0016651 | oxidoreductase activity, acting on NAD(P)H | 86 | 1.97 | <0.001 | 0.0050 |
| GO:0008135 | translation factor activity, RNA binding | 83 | 1.85 | <0.001 | 0.019 |
| GO:0099589 | serotonin receptor activity | 20 | 1.78 | 0.0015 | 0.031 |
| GO:0051540 | metal cluster binding | 55 | 1.74 | <0.001 | 0.038 |
| GO:0070003 | threonine-type peptidase activity | 19 | 1.73 | 0.0060 | 0.039 |

# Supplemental Figures


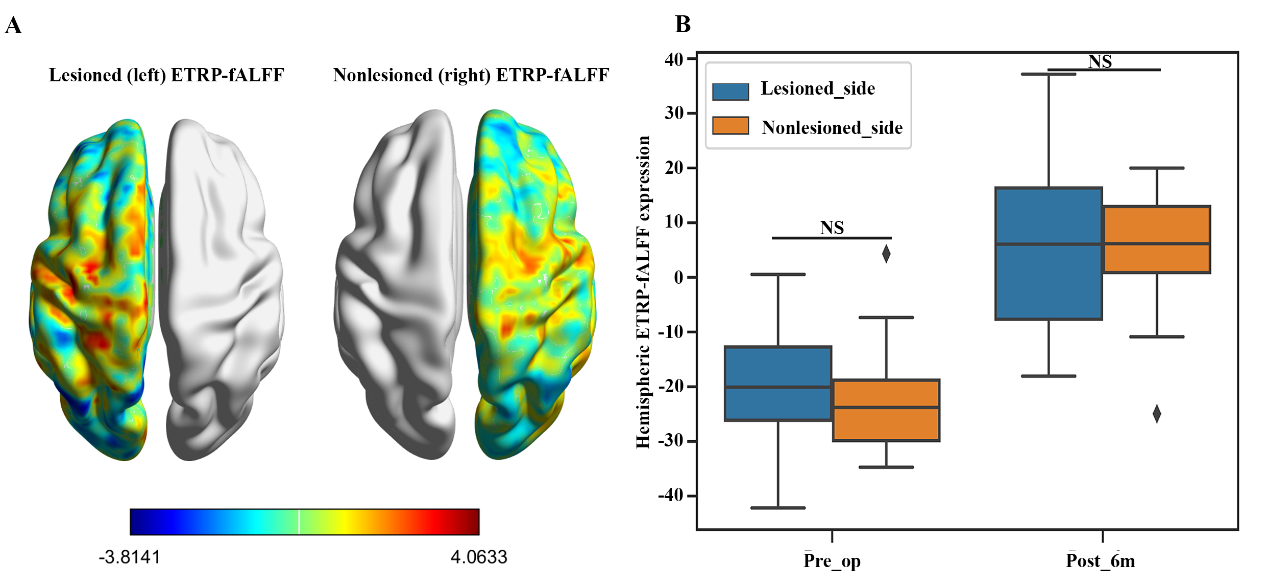


**Figure S1. Hemispheric ETRP-fALFF.** (A) Significant hemispheric ET-related networks identified using OrT/CVA for each hemisphere. (B) No significant difference in the hemispheric ETRP-fALFF expression between lesioned and non-lesioned sides at pre-operation and postoperative 6-month.


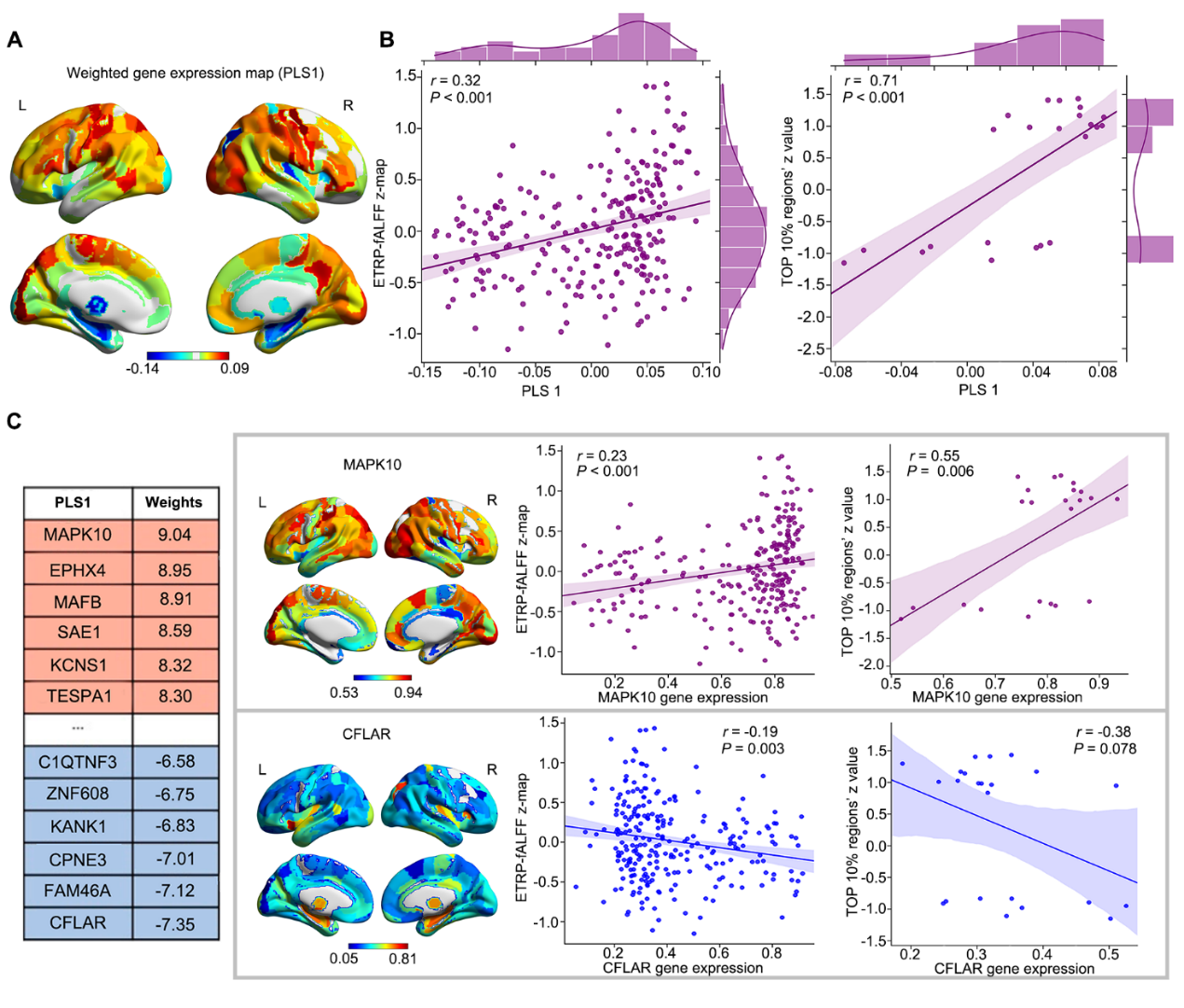


**Figure S2. Gene expression profiles related to ETRP-fALFF z-map.** (A) Cortical map of regional PLS1 scores. (B) Correlation results between PLS1 scores and z values in the global and the top 10% regional level. (C) Genes strongly positively weighted on PLS1 (e.g., MAPK10) were positively correlated with z values in the global and the top 10% regional level, whereas genes strongly negatively weighted on PLS1 (e.g., CFLAR) were negatively correlated with those.

# References

[1] Levi Chazen J, Stradford T, Kaplitt MG. Cranial MR-guided Focused Ultrasound for Essential Tremor : Technical Considerations and Image Guidance. Clin Neuroradiol 2019;29(2):351-7.

[2] Elias WJ, Lipsman N, Ondo WG, et al. A Randomized Trial of Focused Ultrasound Thalamotomy for Essential Tremor. N Engl J Med 2016;375(8):730-9.

[3] Stacy MA, Elble RJ, Ondo WG, Wu SC, Hulihan J. Assessment of interrater and intrarater reliability of the Fahn-Tolosa-Marin Tremor Rating Scale in essential tremor. Mov Disord 2007;22(6):833-8.

[4] Morris SB, DeShon RP. Combining effect size estimates in meta-analysis with repeated measures and independent-groups designs. Psychological Methods 2002;7(1):105-25.

[5] Burt JB, Helmer M, Shinn M, Anticevic A, Murray JD. Generative modeling of brain maps with spatial autocorrelation. Neuroimage 2020;220:117038.
